# Opportunities for and Challenges of Conducting Indirect Treatment Comparisons and Meta-Analyses for Vaccines in Post-EU HTA Regulation Era

**DOI:** 10.3390/jmahp13020031

**Published:** 2025-06-11

**Authors:** Charlotte Graham, Erin Barker, Joe Moss, Emily Gregg, Rachael McCool, Nathalie Largeron, Mélanie Trichard, José Bartelt-Hofer, Maribel Tribaldos

**Affiliations:** 1 York Health Economics Consortium, University of York, York YO10 5NQ, UK; 2 Sanofi, 14 Espace Henry Vallée, 69007 Lyon, France

**Keywords:** indirect treatment comparison, meta-analysis, comparative effectiveness, vaccine, health technology assessment, EU Regulation on Health Technology Assessment, joint clinical assessment

## Abstract

The dynamic nature of infectious diseases introduces inherent challenges to the design of vaccine clinical trials, which consequently makes vaccine indirect treatment comparisons (ITCs) and meta-analyses (MAs) more challenging compared with regular pharmaceuticals. However, comparisons of efficacy and safety between vaccines are being frequently required in vaccine decision making due to a low number of head-to-head clinical trials in the vaccine landscape. The introduction of the European Union Health Technology Assessment (HTA) Regulation (EU HTAR) aims to harmonize HTA efforts across Europe. However, the EU HTAR could also escalate existing challenges for conducting vaccine MAs and ITCs. Such challenges include generating efficacy evidence in time for Joint Clinical Assessment (JCA), incorporating high levels of heterogeneity due to infectious disease-specific characteristics, and tackling a high number of PICOs per submission—likely driven by heterogeneity in the available data and differences in national vaccine calendars. Opportunities to tackle these challenges include introducing a stepwise approach to vaccine assessment in JCA, best-practice recommendations for conducting/interpreting vaccine MAs and ITCs, and condensing the number of PICOs to create larger ‘catch-all’ ITC networks. This perspective article explores these challenges and opportunities further.

## 1. Introduction

Vaccines are one of the most important public health interventions—and health technologies—worldwide, having saved an estimated 154 million lives in the past 50 years [1]. Unlike most health technologies, vaccines can be used for disease prevention (prophylaxis) and are administered to healthy individuals, including children. Consequently, vaccines are subject to more complex and intricate decision making processes, and they must be added to a country’s National Immunization Program (NIP) before being administered to the target population(s).

Health technology assessment (HTA) is a multidisciplinary and systematic process used to inform decision making by determining the clinical and economic value of a health technology. However, the HTA process is oriented towards medicines, and dedicated HTA bodies often have limited experience in applying HTA methodologies to vaccines. Instead, vaccine assessment is typically conducted by National Immunization Technical Advisory Groups (NITAGs), which are vaccine-specialist organizations that provide evidence-based recommendations concerning the inclusion of a vaccine in the NIP and assess vaccines in all European Union (EU) member states [2]. Of the 27 member states in the EU, 12 may also involve an HTA body in the vaccine assessment process. However, 75% of these 12 member states have no formal vaccine decision-analysis framework, which means that vaccine assessment is similar to medicines and does not account for vaccine specificities [2].

HTA decision making in the EU—for medicines and vaccines alike—has been historically made at a national level, with limited cooperation between member states. New legislation called the ‘EU Regulation on HTA’ (EU HTAR) [3] was adopted in December 2021 with the aim of consolidating and harmonizing HTA processes and clinical assessment across all member states. However, conclusions regarding the overall clinical added value of the technology will remain at a national level. The EU HTAR will be applied in a staggered manner, beginning with cancer medicines and advanced therapy medicinal products in January 2025 and extending to orphan drugs from January 2028. All other EU-centrally approved medicines (including vaccines) will be included in the scope of the legislation from January 2030.

It is hoped that the EU HTAR process will improve access to innovative health technologies across Europe, reduce the duplication of HTA efforts between countries, and improve resource efficiency. The EU HTAR could also help to address the substantial delay that occurs between the licensing of a vaccine and its population access in the EU [4]. However, it has been suggested that applying the same processes for clinical assessment for both medicines and vaccines could limit the EU HTAR’s effectiveness, contributing to delayed or limited access to new vaccines [5]. Recital 24 and Article 4 of the legislation [3] acknowledge the specificities of vaccines. However, methodological and procedural guidelines published to date do not discuss vaccine specificities, and possible relationships between the HTA Coordination Group (HTACG) and NITAGs have not yet been defined. As vaccines will not be assessed under the EU HTAR until 2030, there will be time to address the specificities of vaccine assessment in the EU HTAR. For example, including NITAG members that have vaccine-specific competencies and experience in considering the broad health and socioeconomic impact of vaccines.

A key element of the EU HTAR is Joint Clinical Assessment (JCA). This will primarily consider the relative clinical effectiveness and safety of the health technology, with the evaluation of economic evidence and decisions regarding price and reimbursement remaining at a national level within each member state. The clinical evidence presented in the JCA dossier will be synthesized via a systematic literature review (SLR). Where sufficient direct evidence is identified, a meta-analysis can be used to combine the results of multiple studies, increasing the sample size and allowing for greater precision in detecting clinically meaningful effects. Conversely, indirect treatment comparison (ITC) methods can be used to compare vaccines that have not been directly evaluated in a head-to-head trial.

There is a growing need for vaccine ITCs because head-to-head comparisons are often unavailable. This is partly due to a crowded vaccine landscape, where there are increasing numbers of vaccines being developed to target similar diseases and populations, as well as smaller sample sizes within vaccine randomized controlled trials (RCTs, the gold standard study design for evaluating safety and efficacy). Additionally, to ensure that the JCA is relevant across the EU, each member state will be required to complete a PICO (population, intervention, comparator, outcomes) survey. It is thereby anticipated that technologies assessed under the EU HTAR will be subject to a large number of PICOs, introducing greater diversity in study designs, populations, and outcomes. The more diversity included in an evaluation, the more likely indirect comparisons will be required. Despite this, vaccines are associated with unique challenges that make these types of analyses inherently more difficult. Consequently, there are few published examples of MAs and ITCs [6] for vaccines in the literature. In preparation for JCA, the HTACG recently adopted practical and methodological guidelines on quantitative evidence synthesis for both direct and indirect evidence [7]. While this describes the strengths and weaknesses associated with MA and different ITC methods, it does not acknowledge the specific challenges of performing MAs/ITCs for vaccines. To the authors’ knowledge, no guidelines for conducting these statistical methods for vaccines exist.

A previous study by Vaccines Europe identified MAs and ITCs as one of the anticipated challenges for the JCA of vaccines. Therefore, this perspective article presents an in-depth exploration into the challenges of conducting MAs and ITCs for vaccines in light of the EU HTAR and describes potential opportunities for overcoming these challenges, based on the expert opinions of vaccine industry and statistical experts after consulting the proposed guidelines and current literature on the subject matter. Furthermore, while the focus of this article is on vaccines, some of the challenges identified may be applicable to pharmaceuticals or other medical devices.

## 2. Challenge 1: Data Availability at the Time of JCA Dossier Submission

**Figure 1:**
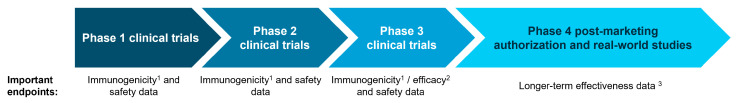
The type of evidence available throughout the lifecycle of a vaccine. ^1^ Immunogenicity data measure the immune response elicited by a vaccine [12]. ^2^ Efficacy data describe whether the vaccine produces the expected result under ideal conditions [13]. ^3^ Effectiveness data measure the degree of beneficial effect within real-world settings [13].

Guidance issued by the HTACG [8] confirms that the clinical evidence presented in the JCA dossier may include a range of studies, from pivotal RCTs to studies that collect real-world evidence (RWE) (Figure 1). RCTs are the gold standard for collecting clinical evidence on safety and efficacy because the randomization eliminates much of the biases that are inherent to other study designs. However, the full impact and potential benefits of a vaccine are often not captured in RCTs [5]. This is partly due to ‘healthy bias’ in the vaccine RCT population [9], and because the ‘effectiveness’ of a vaccine depends on continuously evolving attack rates, antigenic variation, and the vaccine coverage rate—which is only captured in real-world and Phase 4 post-marketing authorization studies years after the vaccine has been introduced to the general population. While RWE is useful for capturing longer-term data, it is associated with higher levels of inherent bias and should, therefore, complement and not replace RCT evidence. Real-world studies may also require long time horizons. The World Health Organization recognizes that RWE plays an important role in vaccine assessment and explicitly recommends using at least five years of data to estimate the existing disease burden for influenza [10]. It is also very challenging to capture the full impact and potential benefits of a rare vaccine in an RCT (for example, Japanese Encephalitis [11]); this would require a long study duration and a large number of participants—something more suited to a real-world study.

Vaccine development typically begins with studies focusing on immunogenicity and safety before progressing to those assessing efficacy. As a result, efficacy data may not be available at the time of JCA submission, leading to a reliance on early clinical trial (immunogenicity) data. This level of data may, in certain scenarios, suffice for full vaccine approval—acting as surrogates of protection [14,15]. However, it more commonly results in ‘conditional’ approval pending the delivery of Phase 3 efficacy data [16]. This approach has previously been used to approve, for example, COVID-19 vaccines in 2020 [17] and a 20-valent pneumococcal conjugate vaccine in 2022 [18]. Surrogate endpoints are particularly important in studies against vaccine-preventable infections (e.g., diphtheria and tetanus) where there are very few cases remaining due to routine vaccine schedules. In these scenarios, it is very challenging to directly measure efficacy, and immunologic measures (accepted as surrogates of protection) ultimately play a larger role in vaccine assessment. However, there is often little consistency in the surrogates of protection used between trials, which makes it challenging to form direct or indirect comparisons. While the HTACG has issued guidance on outcomes for JCA [19], this does not provide guidance on how to address heterogeneity in surrogate endpoints.

An additional approach that the HTACG could consider is the feasibility of first conducting vaccine MAs/ITCs when Phase 3 efficacy data are available, and later be complemented with stand-alone MAs/ITCs of observational data that are reflective of mature RWE [5]. While this would help to address the challenge of limited data availability at the time of product launch, there may still be challenges regarding the heterogeneity of data (as discussed further in Challenge 2). In this scenario, the HTACG guidance on the validity of different studies [8] is beneficial as it reinforces how RWE should be complementary to, rather than in place of, clinical trial data.

**Figure 2:**
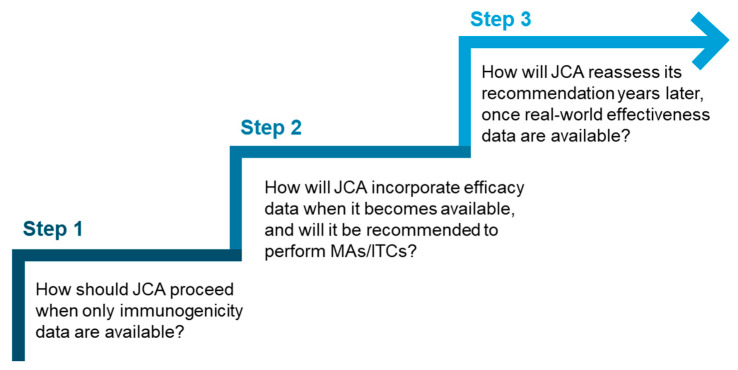
Key questions that could inform a stepwise approach for vaccine assessment in JCA.

Given the potential variation in the extent of data available at the time of JCA submission, it would be beneficial for the HTACG to introduce a stepwise approach to vaccine assessment. This approach could offer different levels of guidance based on the data available at the time of submission and answer key questions (Figure 2), for example, at what stage MAs and ITCs should be conducted. This could also be supplemented by best-practice recommendations or a framework for conducting and interpreting MAs and ITCs for vaccines.

## 3. Challenge 2: Heterogeneity Due to Infectious Disease-Specific Characteristics

The dynamic nature of infectious diseases also introduces inherent challenges to the design of vaccine clinical trials, which consequently makes the development and interpretation of vaccine MAs and ITCs more challenging. Factors such as seasonality, geographical epidemiology, and antigenic variation contributing to the creation of new strains of disease introduce additional layers of complexity that are not typically encountered with standard pharmaceuticals. These characteristics affect the assessment of vaccines two-fold: (1) they can lead to seasonal-dependent data that are frequently heterogeneous over time, and (2) they can impact the efficacy and effectiveness of a vaccine. During JCA dossier development, it is important that the dynamic nature of infectious diseases is recognized and the impact on MAs/ITCs is acknowledged.

Firstly, it is important that the SLR (by which the evidence for the MA/ITC was identified) is robust in its methodology. A feasibility assessment should be conducted to identify the key sources of heterogeneity across the studies and to facilitate the selection of the most appropriate method(s) to analyze the data. While this process should be implemented for any health technologies or pharmaceuticals, the unique nature of heterogeneity for infectious diseases can lead to complex networks and comparisons that may be misinterpreted by decision makers. NITAGs bring valuable vaccine expertise that can help to accurately interpret these complex networks. However, to date, the relationship between NITAGs and the HTACG during the JCA process remains undefined. This lack of clarity could hinder the integration of NITAG expertise into the EU HTAR framework (and any associated guidance documents).

An example of where heterogeneity has limited the extent to which analysis is possible is in the recent European Centre of Disease Control (ECDC) SLR of the safety, efficacy, and effectiveness of influenza vaccines [20]. In this report, some MAs were deemed unfeasible due to the extent of heterogeneity, with none of the planned subgroup analysis (e.g., strain, clade, and season) possible. While this SLR was highly anticipated, the overall reliability of the SLR has also been critiqued [21,22]. Specifically, there have been concerns regarding the determination of evidence using the GRADE methodology and the reproducibility of the report [22]. This could influence its practical implementation, particularly if it were to be used to inform decision making. Following EU HTAR implementation, Joint Scientific Consultation will be an important stage to ensure that vaccine clinical trial design is suitable and to reduce bias. However, these early engagements cannot predict the circulation of strains/seasonality, nor foresee challenges faced at data analysis/outcome interpretation stages.

Secondly, antigenic variation can contribute to the appearance of new strains and can affect the virulence of disease, and the outcomes of some vaccines have been shown to vary in different strains [23,24,25]. This introduces further heterogeneity that could be amplified in the EU HTAR, where all 27 member states will be included in the evaluation. Due to the wide geographical spread of JCA, it is likely that different disease strains will be relevant in different countries—and this will change year to year. If the aim of JCA is to reflect an EU-wide population, this does not cause additional challenges. However, each member state is required to give due consideration to the final JCA report, which should inform national HTA process (even though it is not legally binding) [3]. Therefore, it is important for national-level decision makers to ensure that the data are representative of the member state population and, if not, consider what impact this may have.

## 4. Challenge 3: Harmonizing Vaccination Strategies and Priorities Across Europe in the Context of Multiple JCA PICOs

A key value proposition of the EU HTAR is that it is intended to reduce the duplication of HTA efforts and improve the availability of health technologies across the EU. However, this means that the HTACG is inevitably presented with the challenge of consolidating all PICO preferences into the minimal number of PICOs that still covers the need of all 27 member states. It is likely that the key differences in PICOs between member states will be the target population(s) and available comparator(s)—which will partly be driven by differences in vaccine calendars between member states. Furthermore, the standard of care that is available at the launch of a new vaccine may not be available at the time of the Phase 3 clinical trial. Therefore, comparators used in an RCT setting may not be the most relevant comparators expected by the HTACG at the time of JCA submission.

It is unclear how the EU HTAR will approach any differences in populations and comparators. One method would be to require that the populations/comparators for each member state must be exactly captured in one of the final PICOs. This would mirror the approach used by the National Institute for Health and Care Excellence (NICE) in the UK, where comparators only directly relevant to the UK are included. If the EU HTAR implements this approach, it will likely increase the number of anticipated PICOs. This would infer a high burden on HTDs to ensure that all MAs, ITCs, sensitivity analyses, and subgroup analyses are completed within the narrow 100-day JCA submission period [26].

Alternatively, the EU HTAR could try to condense the number of PICOs by grouping populations and comparators together, if applicable. This would create larger ‘catch-all’ ITC networks that leverage a high number of populations/comparators, capture the populations/comparators for all member states in fewer PICOs, and alleviate some demand on the HTDs. However, not all therapies included in a network would be relevant to all member states, and the network may be large and difficult to navigate. This, therefore, will increase the risk that the MA/ITC results will be misinterpreted by decision makers.

While the above challenges may be applicable to any pharmaceutical or health technology being assessed through JCA, it is additionally challenging for vaccines because of the increased heterogeneity in surrogate endpoints (immunogenic outcomes) at the time of JCA, time-dependent outcomes, and an increased use of RWE over RCTs. Different procurement methods could also amplify differences in NIPs and, therefore, comparators between member states. It is true that a proportion of newly developed vaccines focus on infectious diseases where there are not many comparators. However, a large number of vaccine candidates are being developed within similar therapeutic areas, contributing to a crowded vaccine space. For example, five COVID-19 vaccines are available in the EU, with a further eight treatments available [27]. Furthermore, there are three vaccines for respiratory syncytial virus (RSV) either approved or in development in the EU—in addition to prophylaxis medicines [28,29,30]. These treatments also span several populations, including pregnant women, children, and older adults.

Due to these challenges, it will be important for the member states’ NITAG to be involved in submitting the national PICO preferences. This will help ensure that the final PICOs are tailored to vaccines. It is likely that the NITAG for each member state will be working on nationwide recommendations for the vaccine at the same time as the JCA to ensure efficient and rapid implementation of the vaccine once available. This parallel process introduces risks that the final JCA report will not be fit for purpose given that, in some countries, the NITAG may not have finalized and published its recommendation before the JCA process.

## 5. Opportunities to Resolve These Challenges

**Figure 3:**
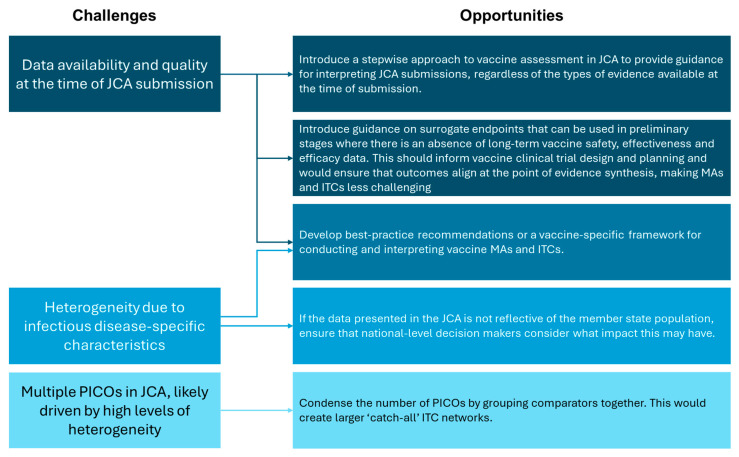
Summary of the challenges and opportunities for vaccine MAs and ITCs in light of the EU HTAR.

Vaccine assessment presents several challenges, including in the conduct of MAs and ITCs. However, the implementation of the EU HTAR provides a unique opportunity to resolve these issues (Figure 3). This includes creating new—or revising existing—methodological guidelines to explicitly acknowledge vaccine specificities, referring to key references developed by the WHO and EMA [14,31]. Of note, recommendations for data collection and the use of surrogate endpoints in early clinical trials should be included, and a relationship between the HTACG and NITAGs should be outlined. A dedicated framework for conducting vaccine MAs and ITCs would also be beneficial; existing guidance caters to traditional pharmaceuticals and health technologies, and there is a paucity of literature addressing these types of statistical analyses for vaccines. Condensing the number of PICOs within each JCA would also assist in creating larger ‘catch-all’ ITC networks. This would reduce the burden on HTDs for conducting statistical analyses, but may be more challenging for decision makers to interpret. As vaccines will not be included in the scope of JCA until 2030, there remains time to address these concerns and optimize future vaccine assessment.

## 6. Conclusions

Direct comparisons of vaccines in the context of double-blinded RCTs will remain the gold standard for defining clinically meaningful differences, with no exception for vaccines. However, ITCs are being more frequently required in vaccine assessment because there are few direct comparisons of vaccines. It is also anticipated that technologies assessed under the EU HTAR will be subject to a large number of PICOs. This increases the likelihood that indirect comparison will be required because more diverse study designs, populations, and outcomes are introduced, therefore making direct comparisons more challenging. The introduction of the EU HTAR provides an opportunity to standardize the methods for vaccine assessment across the EU, including creating best-practice recommendations or a vaccine-specific framework for conducting and interpreting vaccine MAs and ITCs. While this is promising, the EU HTAR could also escalate existing issues for performing vaccine ITCs. For example, generating evidence of efficacy in time for HTA will remain challenging because the full and indirect benefit of the vaccine—especially its effectiveness—is frequently demonstrated with delay, particularly in the years following marketing authorization and reimbursement.

## Data Availability

No new data were created or analyzed in this study. Data sharing is not applicable to this article.
